# The Nomogram for Early Death in Patients with Bone and Soft Tissue Tumors

**DOI:** 10.7150/jca.46152

**Published:** 2020-07-09

**Authors:** Yao Xu, Guijun Xu, Haixiao Wu, Feng Lin, Min Mao, Vladimir P. Baklaushev, Vladimir P. Chekhonin, Karl Peltzer, Jun Wang, Guowen Wang, Xin Wang, Chao Zhang

**Affiliations:** 1 Department of Bone and Soft Tissue Tumors, Tianjin Medical University Cancer Institute and Hospital, National Clinical Research Center for Cancer, Key Laboratory of Cancer Prevention and Therapy, Tianjin's Clinical Research Center for Cancer, Tianjin, China.; 2 Department of orthopaedics, Tianjin Hospital, Tianjin, China.; 3 Department of Pathology and Southwest Cancer Center, First Affiliated Hospital, Army Medical University, Chongqing, China.; 4 Federal Research and Clinical Center of Specialized Medical Care and Medical Technologies, Federal Biomedical Agency of the Russian Federation, Moscow, Russian Federation.; 5 Department of Basic and Applied Neurobiology, Federal Medical Research Center for Psychiatry and Narcology, Moscow, Russian Federation.; 6 Department of Research and Innovation, University of Limpopo, Turfloop, South Africa.; 7 Department of Oncology, Radiology and Nuclear Medicine, Medical Institute of Peoples' Friendship University of Russia, Moscow, Russia.; 8 Department of Epidemiology and Biostatistics, First Affiliated Hospital, Army Medical University, Chongqing, China.

**Keywords:** Bone and soft tissue tumors, SEER program, Early death, Nomograms

## Abstract

**Objectives:** The present study aimed to evaluate the early mortality rate and associated factors for early death in bone and soft tissue tumors, and to construct predictive nomogram.

**Methods:** Patients diagnosed between 2010 and 2015 in the Surveillance, Epidemiology, and End Results (SEER) dataset were enrolled. The early death (survival time ≤ 3 months) rate was calculated and associated risk factors were evaluated by the logistic regression models. The significant factors were used to construct predictive nomograms.

**Results:** A total of 2,003 (8.5%) patients died within 3 months after cancer diagnosis, among whom 1,146 (4.9%) patients died from cancer-specific cause. Older age (19-50 and >50 years), grade (III and IV), reginal or distant stage were associated with higher odds of total, cancer-specific and non-cancer-specific early death. T2 stage, metastasis to brain and lung were risk factors for total and cancer-specific early death. Surgical interventions significantly decreased the odds of total, cancer-specific and non-cancer-specific early death. Female and black race were associated with lower odds of non-cancer-specific early death. The area under the curve (AUC) of the nomograms for total early death, cancer-specific and non-cancer-specific early death prediction was 88.0%, 89.0% and 83.2%, respectively.

**Conclusions:** A total of 8.5% patients with bone and soft tissue tumors suffered early death. Several risk factors were associated with higher odds of early death while surgery can decrease the possibility of early death. Nomograms based on all related factors can be used to estimate the early death in bone and soft tissue tumors.

## Introduction

Bone and soft tissue tumors have received expanding attention in the latest years. According to the latest cancer statistics in United States 2019, the estimated new cancer cases and deaths for bones, joints and soft tissue were 16,250 and 6,930 cases, respectively 1. Data from other countries showed the age-standardized incidence in soft tissue sarcoma was 2.8-4.5/100,000 person-years while it was 0.2-2.1/100,000 for bone sarcoma 2-4. As to the mortality, the age-standardized rate in Switzerland from 2011 to 2015 was 1.42 per 100,000 person-years in soft tissue sarcoma and 0.42 in bone sarcoma 3. The Shanghai Cancer Registry during 2002-2014 revealed that the mortality rate was 1.0 per 100,000 and 0.2 per 100,000 person-years in soft tissue and bone sarcomas, respectively 4.

With the development of treatment strategies, the survival of patients with bone and soft tissue tumors has been significantly improved. In the previous study, the five-year relative survival of soft tissue sarcomas reached to 61.6% (95%CI 58.6-64.4) and it was 73.1% (95%CI 66.6-78.6) in bone sarcomas 3. To improve the long-term survival, potential risks of complications were investigated in Ewing sarcoma 5. A prognostic model was further established to predict the 5-year overall survival of patients with extremity osteosarcoma 6. However, seldom study looked into the patients who died early after diagnosis. Patients with high risk of early death should be timely identified so that individualized treatments and/or supportive care can be scheduled to improve their survival and life quality.

A previous study prospectively reported 595 (12%) cases died within 1 year after diagnosis of bone and soft tissue sarcoma 7. In another study regarding to the advanced soft tissue sarcoma, authors carried out a retrospective analysis and developed a prognostic model for early death within 3 months 8. The performance status and distant metastasis were retained as risk factors by logistic regression analysis in the study. The rates of early death were 8.6% and 4.5% in two cohorts of soft tissue sarcoma. Higher rate of early death was noticed in patients with more risk factors 8. In the latest study based on 2,432 osteosarcoma and 1,619 Ewing sarcoma cases aged from 0 to 49 years, the average mortality rate at 3 months was 1.7% and 2.0% in osteosarcoma and Ewing sarcoma, respectively. The mortality rate at 6 months was similar to that at 3 months 9. These studies provided us the insight into the early mortality. However, to our knowledge, there were no studies thoroughly investigating the 90 days mortality in bone and soft tissue tumors. The early death predictive model in bone and soft tissue tumors was needed.

Based on a large cohort from Surveillance, Epidemiology, and End Results (SEER) datasets, the current study was conducted to evaluate the incidence of early death in patients with bone and soft tissue tumors. According to the previous literature, the early death was defined as overall survival time ≤ 3 months after initial diagnosis 8. Related risk factors were further identified and the factors-based nomogram was constructed to predict the early death.

## Materials and Methods

### Ethics statement

Cancer is a reportable disease in every state of the USA and the data in the SEER database does not need informed patient consent. The present study was complied with the 1964 Helsinki Declaration and its later amendments or comparable ethical standards.

### Data source and cohort selection

**Figure 1:**
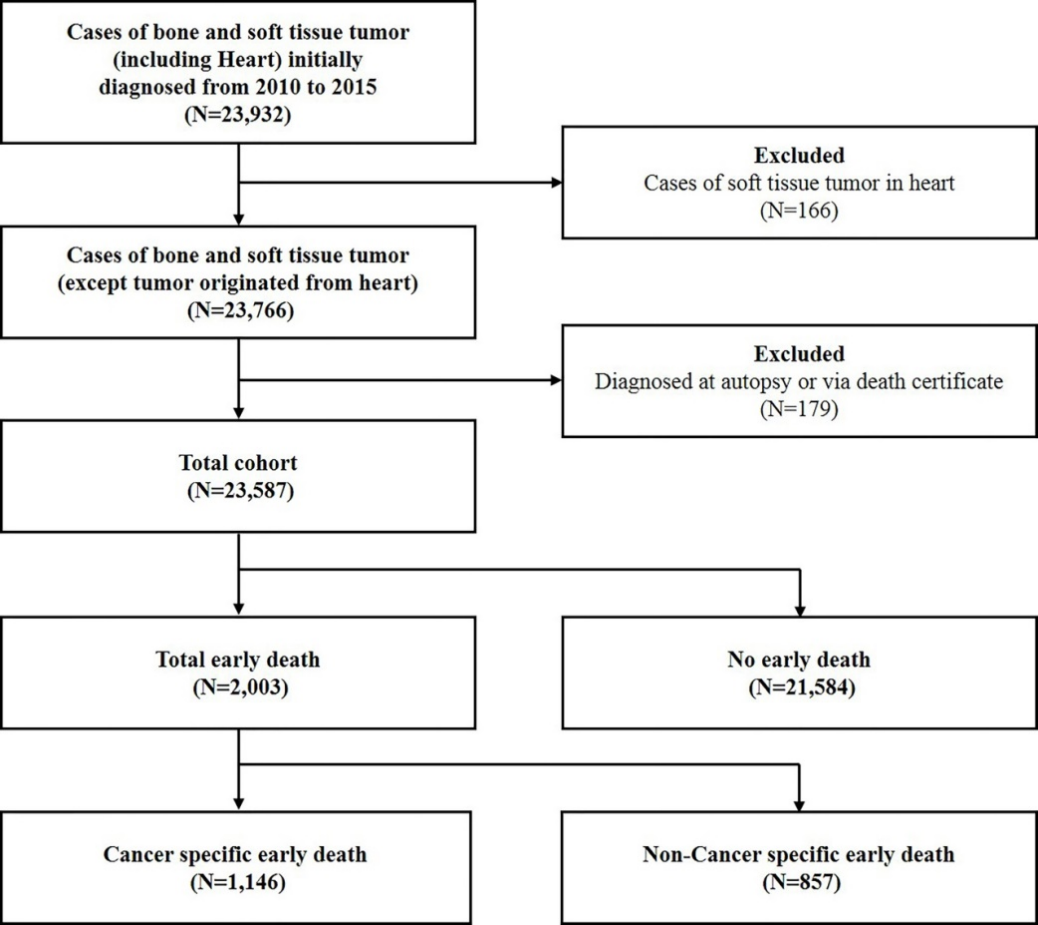
Flow-chart for patient selection with bone and soft tissue tumor.

The SEER database (https://seer.cancer.gov/) is compiled by the National Cancer Institute and covers approximately 28% of the population in USA. The data used in the present study was obtained from the latest version by April 2019, which was named as Incidence - SEER 18 Regs Research Data + Hurricane Katrina Impacted Louisiana Cases, Nov 2018 Sub (1975-2016 varying). The flow-chart of the selection criteria was listed in Figure 1. Due to the low incidence and unfavorable prognosis, patients with soft tissue tumor originated from heart were excluded from the present study 10,11. Meanwhile, patients diagnosed at autopsy or via death certificate were excluded. A total of 23,587 patients with bone and soft tissue tumors diagnosed between 2010 and 2015 were included.

### Statistical analysis

Patient demographic and clinical characteristics included age at diagnosis (≤18, 19-50 and >50 years), gender (Male and Female), marital status (Married and Unmarried), race (White, Black and Others), insurance (Insured and Uninsured), primary tumor site (Bone or Soft tissue), location of tumor (Extremities, Head and neck, or Trunk), tumor differentiation grade (Grade I, II, III and IV), laterality (Left-sided, Right-sided, One side NOS or Paired sides), SEER stage (Localized, Regional and Distant), T stage (T1, T2 and T3), N stage (N0 and N1), surgery treatment (No surgery, Local surgery, Partial surgery, Radical surgery or Amputation). The presence or absence of metastasis to bone, brain, liver and lung were also included. The patients without specific information about aforementioned variables, which listed as “Unknown” group, were also included in our analysis and final nomogram construction.

The primary outcome in this study was all-cause, cancer-specific and non-cancer specific early death. Variables (with *P*<0.05) in univariable logistic regression were further analyzed using a multivariable logistic regression model. Significant variables in the multivariable logistic regression model were included in the construction of the predictive nomograms for all-cause, cancer-specific and non-cancer early death. The performance of the nomogram was evaluated by the receiver operating characteristics (ROC) and the greater area under the curve (AUC) close to 1.0 indicated more perfect ability of discrimination. To evaluate the calibration of the nomogram, the calibration plots were produced by bootstrapping with 1,000 resamples. The relationship between the observed and predicted probabilities of all-cause, cancer-specific and non-cancer early death was described graphically.

All statistical tests were two-sided, and *P*<0.05 was considered significant. All the statistical analyses were conducted by the IBM Statistical Package for the Social Sciences (SPSS) version 23.0 software package for Windows (SPSS, Inc. Chicago, IL, USA).

## Results

### Clinical characteristics and incidence of early death

A total of 2,003 (8.5%) patients were defined as early death, among those 1,146 (4.9%) patients died due to cancer-specific cause while others were non-cancer-specific cases. The incidence of early death was 6.5% (324/4,957) and 9.0% (1,679/18,630) for bone and soft tissue tumors, respectively. Most patients with early death were white race (80.5%), insured (89.5%) and diagnosed after 50 years old (87.1%). As to the tumor location, the most common location was trunk (40.5%), followed by extremities (24.7%). Except for the unknown grade (46.5%), grade IV (28.5%) and III (17.4%) were the common types in early death patients. Besides, a total of 1,455 (72.6%) patients did not receive any surgical intervention in early death group. Basic characteristics for patients without early death, with total early death, with cancer-specific early death and with non-cancer-specific early death were summarized in Table 1.

### Risk factors for early death

Older age (19-50 and >50 years), tumor originated from soft tissue, tumor in head and neck or trunk, higher tumor differentiation grade, one side NOS and paired sides, reginal or distant stage, higher T stage, N1 stage, and metastasis to bone, brain, liver and lung were associated with higher odds of total, cancer-specific and non-cancer-specific early death in univariable logistic regression. Black race and uninsured status were related with higher odds of cancer-specific early death. On the contrary, surgical interventions significantly decreased the odds of early death. Besides, female, black and other races were associated with lower odds for non-cancer-specific early death. The detail information was listed in Table 2.

The Multivariable analysis was summarized in Table 3. Older age (19-50 and >50 years), higher tumor differentiation grade (III and IV), reginal and distant stage were associated with higher odds of total, cancer-specific and non-cancer-specific early death. T2 stage, metastasis to brain and lung were risk factors for total and cancer-specific early death. On the contrary, surgical intervention significantly decreased the odds of total, cancer-specific and non-cancer-specific early death. Female and black race were associated with lower odds for non-cancer-specific early death.

### Performance of the nomogram for predicting early death

According to the results in the multivariable logistic regression model, the following variables were selected in the construction of the predictive nomograms for total early death: age, gender, race, tumor location, tumor differentiation grade, laterality, SEER Stage, T stage, brain metastasis, lung metastasis and surgery. The same methods were used for selecting variables in the construction of nomograms for cancer-specific and non-cancer-specific early death.

**Figure 2:**
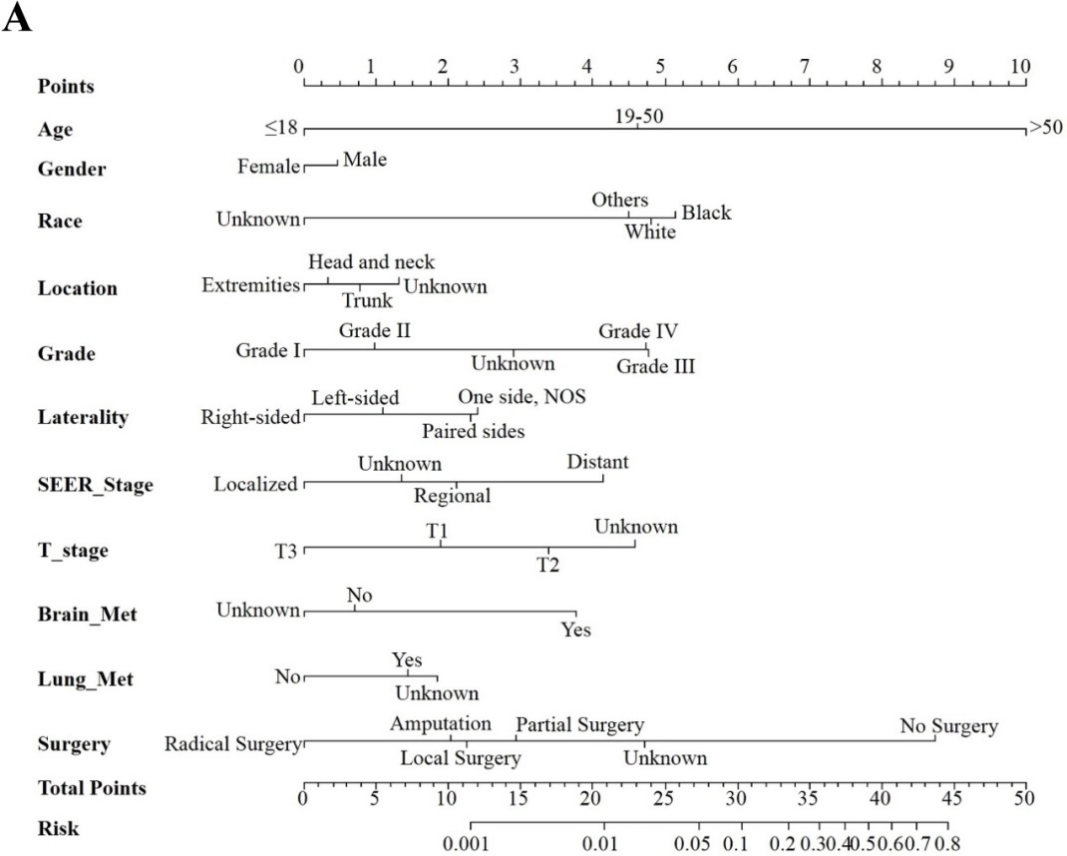

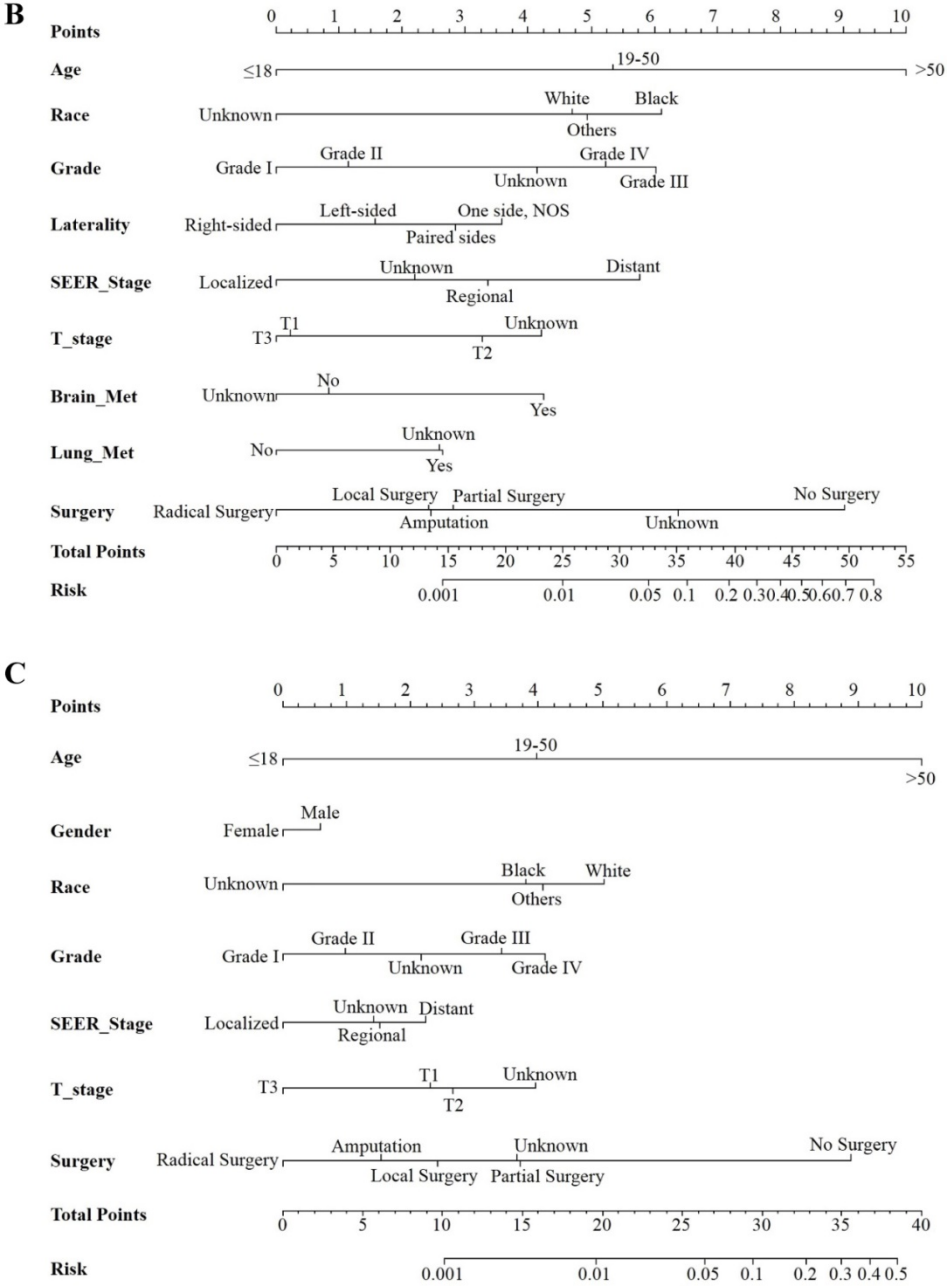
Nomogram for predicting all cause early death (Figure 2A), cancer specific early death (Figure 2B) and non-cancer early death (Figure 2C) in patients with bone and soft tissue tumor.

**Figure 3:**
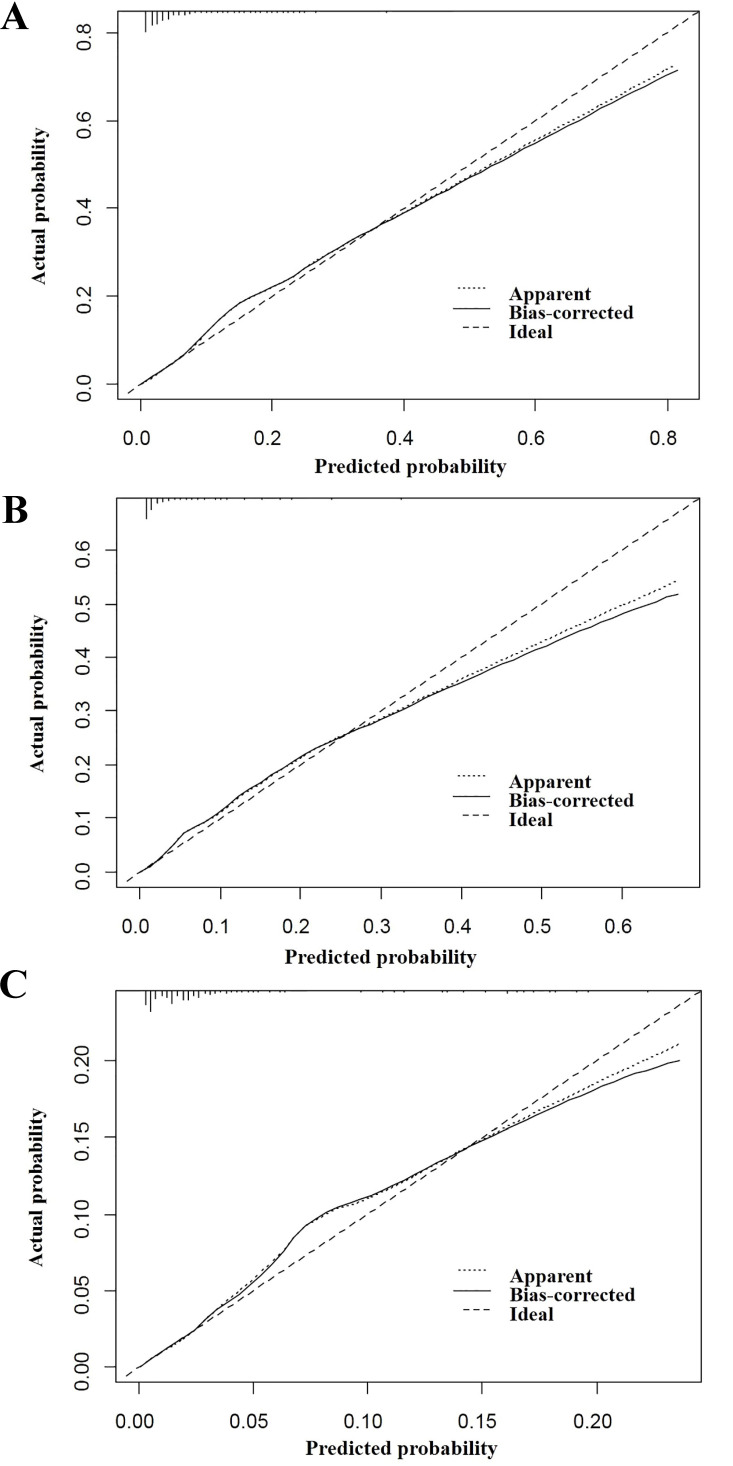
The calibration curve for assessing the calibration of the nomogram in predicting all cause of early death (Figure 3A), cancer-specific early death (Figure 3B) and non-cancer early death (Figure 3C).

**Figure 4:**
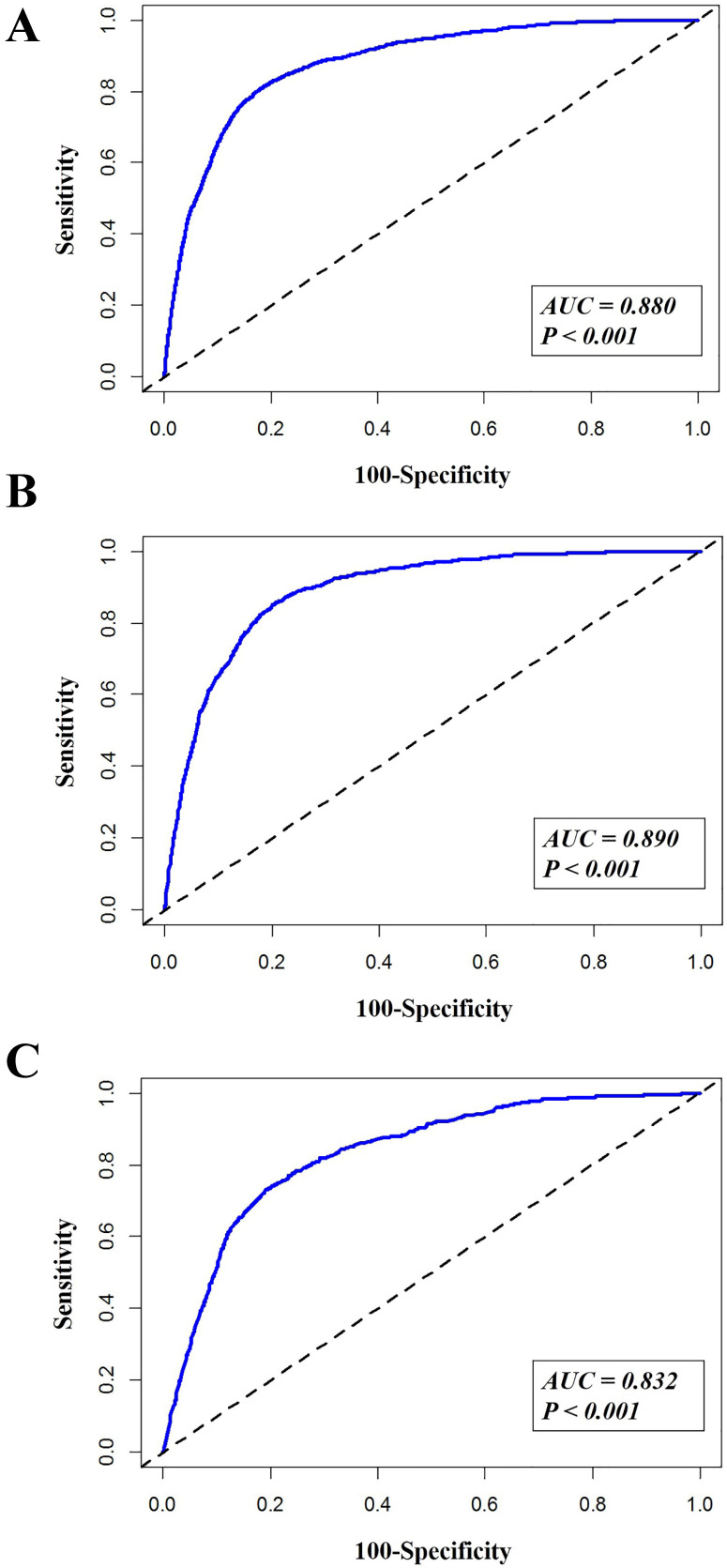
The ROC curve for assessing the discrimination of the nomogram in predicting all cause of early death (Figure 4A), cancer-specific early death (Figure 4B) and non-cancer early death (Figure 4C).

Three nomograms were constructed to predict the odds of total early death, cancer-specific and non-cancer-specific early death among bone and soft tissue tumor patients, respectively (Figure 2A-C). The calibration curve revealed proper agreement between the predicted and observed probabilities in three predictive models with all calibration curves close to the 45-degree line (Figure 3A-C for total, cancer-specific and non-cancer specific early death, respectively). In addition, the AUC of the three nomograms for total, cancer-specific early death and non-cancer-specific early death prediction were 88.0%, 89.0% and 83.2%, respectively. All nomograms showed satisfactory strength of discrimination (Figure 4 A-C).

## Discussion

Early death investigation is crucial to improve the survival of the patients with bone and soft tissue tumors. Based on a large population in the SEER database, it was the first time that the rate and related risk factors for early deaths were evaluated in bone and soft tissue tumors. A total of 16.0% patients suffered early death after the diagnosis of bone and soft tissue tumors in United States from 2010 to 2015. The rate of early death was extremely higher than that of the previous study between 1985 and 2008 9. The significant different early death may be derived from different composition of sample from different regions. The high percentage of early death suggested the physicians offer further attention for the patients with high risk.

Older age was found to be related with higher odds of early death in the present study, especially for the non-cancer specific early death with age older than 50 years. As reported in the previous study, age older than 19 years was an independent factor of early death at 3 months in patients with osteosarcoma 9. Increased negative influence of older age was also revealed in other studies. In studies evaluating the cut-off age of 40 or 50 years for osteosarcoma, older patients showed worse survival 12,13. Besides, the tumor in trunk was more commonly found in older patients and was associated with worse survival in the previous studies and our analysis. Older patients who showed axial tumors and worse survival were revealed in the Ewing sarcoma 14 and soft tissue sarcoma 15. The difficulty of complete resection of tumors in axial trunk may be the potential reason leading to early death. Based on the results in the present study for patients older than 50 years, non-cancer specific causes to death should not be neglected.

Social characteristics have been studied and some factors showed significant impact on the survival of cancer patient. Previous studies showed that support from the families can help against cancer and unmarried patients experienced worse long-term survival 16. There was a small but independent effect of unemployment on 3-month mortality rate (OR: 1.06, 95% CI 1.01-1.10) for patients with osteosarcoma 9. Low-income patients with bone and soft tissue sarcoma showed decreased survival regardless of disease stage 17. However, these trends were not confirmed in the present study. The influence of the socioeconomic characteristics should be further evaluated.

As to the tumor behaviors, higher tumor differentiation grade (III and IV), reginal and distant stage were associated with higher odds of total, cancer-specific and non-cancer-specific early death. All these serious aggressive factors should be considered in the estimation of early death. Although the negative influence of pulmonary or non-pulmonary metastases on long-term survival was reported in previous studies 14,15,18. In the present study, it was the first time that the correlation between the metastasis to brain or lung and cancer-specific early death was revealed. Thus, evaluation for metastasis was encouraged in the patients with high risk of early brain and/or lung metastases, especially in patients with grade III or IV tumor.

Contrary to the abovementioned risk factors, surgical intervention was found to be significantly correlated with the decreased incidence of total, cancer-specific and non-cancer-specific early death. To improve survival and life quality, surgery has been accepted to be one of the fundamental treatments for bone and soft tissue tumors 19,20. Therefore, aggressive surgery should be encouraged for the eligible patients to decrease the development of early death.

In our previous research, the nomogram integrating related risk factors is a good predicting tool for the estimation of early death in stage IV cancer 21. In the present study looking into the early death in patients with bone and soft tissue tumor, the constructed nomograms were proved to be with satisfactory predictive ability. Thus, such tool can be potentially be applied as the auxiliary screening tool for both physicians and patients. The targeted treatment regimens can be further scheduled for patients with high risk of early mortality.

The present study has some limitations. Many histological types of bone and soft tissue tumors were recorded in the SEER datasets. The risk factor analyses in each type were not thoroughly conducted. Thus, prediction should be performed regarding to specific tumor. Factors such as performance status and other treatments were not investigated in the present study due to the unavailable records in the SEER datasets. The preliminary findings and predictive models should be further externally validated.

In conclusion, based on the large population analysis, the incidence of early death in bone and soft tissue tumor was found to be approximate 16%. Older age (19-50 and >50 years), grade (III and IV) and reginal or distant stage were associated with higher odds of early death. Surgery can decrease the occurrence of early death. The constructed nomograms based on the risk factors can be used to estimate the odds of the early death. Targeted screening and treatment regimens can be considered in the patients with high risk after nomogram evaluation.

## Figures and Tables

**Table 1:** Description of the SEER population for patients with bone and soft tissue tumor

Subject characteristics	Patients No. (%)			
	No early death	Total Early death	Cancer specific early death	Non-Cancer specific early death
**Age**				
≤18	2681 (12.4)	42 (2.1)	28 (2.4)	14 (1.6)
19-50	6330 (29.3)	217 (10.8)	148 (12.9)	69 (8.1)
>50	12573 (58.3)	1744 (87.1)	970 (84.6)	774 (90.3)
**Gender**				
Male	11948 (55.4)	1156 (57.7)	648 (56.5)	508 (59.3)
Female	9636 (44.6)	847 (42.3)	498 (43.5)	349 (40.7)
**Marital status**				
Married	9934 (46)	910 (45.4)	515 (44.9)	395 (46.1)
Unmarried	10134 (47.0)	963 (48.1)	570 (49.7)	393 (45.9)
Unknown	1516 (7.0)	130 (6.5)	61 (5.3)	69 (8.1)
**Race**				
White	17175 (79.6)	1613 (80.5)	888 (77.5)	725 (84.6)
Black	2330 (10.8)	231 (11.5)	159 (13.9)	72 (8.4)
Others	1785 (8.3)	152 (7.6)	95 (8.3)	57 (6.7)
Unknown	294 (1.4)	7 (0.3)	4 (0.3)	3 (0.4)
**Insurance**				
Insured	19930 (92.3)	1793 (89.5)	1004 (87.6)	789 (92.1)
Uninsured	734 (3.4)	72 (3.6)	53 (4.6)	19 (2.2)
Unknown	920 (4.3)	138 (6.9)	89 (7.8)	49 (5.7)
**Primary Site**				
Bone	4633 (21.5)	324 (16.2)	175 (15.3)	149 (17.4)
Soft tissue	16951 (78.5)	1679 (83.8)	971 (84.7)	708 (82.6)
**Location**				
Extremities	10804 (50.1)	495 (24.7)	265 (23.1)	230 (26.8)
Head and neck	3986 (18.5)	389 (19.4)	213 (18.6)	176 (20.5)
Trunk	6104 (28.3)	812 (40.5)	469 (40.9)	343 (40.0)
Unknown	690 (3.2)	307 (15.3)	199 (17.4)	108 (12.6)
**Grade**				
Grade I	2896 (13.4)	62 (3.1)	26 (2.3)	36 (4.2)
Grade II	2794 (12.9)	90 (4.5)	41 (3.6)	49 (5.7)
Grade III	3412 (15.8)	349 (17.4)	211 (18.4)	138 (16.1)
Grade IV	5011 (23.2)	570 (28.5)	303 (26.4)	267 (31.2)
Unknown	7471 (34.6)	932 (46.5)	565 (49.3)	367 (42.8)
**Laterality**				
Left-sided	7579 (35.1)	454 (22.7)	253 (22.1)	201 (23.5)
Right-sided	7397 (34.3)	371 (18.5)	189 (16.5)	182 (21.2)
One side, NOS	6425 (29.8)	1133 (56.6)	679 (59.2)	454 (53.0)
Paired sides	183 (0.8)	45 (2.2)	25 (2.2)	20 (2.3)
**Stage**				
Localized	7579 (35.1)	454 (22.7)	164 (14.3)	243 (28.4)
Regional	7397 (34.3)	371 (18.5)	190 (16.6)	170 (19.8)
Distant	6425 (29.8)	1133 (56.6)	640 (55.8)	315 (36.8)
Unknown	183 (0.8)	45 (2.2)	152 (13.3)	129 (15.1)
**T stage**				
T1	6816 (31.6)	208 (10.4)	84 (7.3)	124 (14.5)
T2	10580 (49.0)	815 (40.7)	477 (41.6)	338 (39.4)
T3	116 (0.5)	9 (0.4)	6 (0.5)	3 (0.4)
Unknown	4072 (18.9)	971 (48.5)	579 (50.5)	392 (45.7)
**N stage**				
N0	18467 (85.6)	1225 (61.2)	661 (57.7)	564 (65.8)
N1	964 (4.5)	205 (10.2)	138 (12.0)	67 (7.8)
Unknown	2153 (10.0)	573 (28.6)	347 (30.3)	226 (26.4)
**Bone Met**				
No	19868 (92.0)	1453 (72.5)	790 (68.9)	663 (77.4)
Yes	765 (3.5)	298 (14.9)	209 (18.2)	89 (10.4)
Unknown	951 (4.4)	252 (12.6)	147 (12.8)	105 (12.3)
**Brain Met**				
No	20547 (95.2)	1682 (84.0)	947 (82.6)	735 (85.8)
Yes	73 (0.3)	58 (2.9)	43 (3.8)	15 (1.8)
Unknown	964 (4.5)	263 (13.1)	156 (13.6)	107 (12.5)
**Liver Met**				
No	20228 (93.7)	1514 (75.6)	830 (72.4)	684 (79.8)
Yes	398 (1.8)	228 (11.4)	159 (13.9)	69 (8.1)
Unknown	958 (4.4)	261 (13.0)	157 (13.7)	104 (12.1)
**Lung Met**				
No	19138 (88.7)	1180 (58.9)	589 (51.4)	591 (69.0)
Yes	1487 (6.9)	561 (28.0)	402 (35.1)	159 (18.6)
Unknown	959 (4.4)	262 (13.1)	155 (13.5)	107 (12.5)
**Surgery**				
No Surgery	3799 (17.6)	1455 (72.6)	865 (75.5)	590 (68.8)
Local Surgery	6360 (29.5)	215 (10.7)	102 (8.9)	113 (13.2)
Partial Surgery	1507 (7.0)	67 (3.3)	31 (2.7)	36 (4.2)
Radical Surgery^*^	8306 (38.5)	169 (8.4)	85 (7.4)	84 (9.8)
Amputation	1114 (5.2)	40 (2.0)	23 (2.0)	17 (2.0)
Unknown	498 (2.3)	57 (2.8)	40 (3.5)	17 (2.0)

*Radical excision or resection of lesion with limb salvage; Abbreviations: SEER = Surveillance, Epidemiology, and End Result.

**Table 2:** The univariable logistic regression for analyzing the risk factors for early death

Subject characteristics	Total early death		Cancer specific early death		Non-Cancer specific early death	
	OR (95% CI)	*P value*	OR (95% CI)	*P value*	OR (95% CI)	*P value*
**Age**						
≤18	Ref	*1.0*	Ref	*1.0*	Ref	*1.0*
19-50	2.19 (1.57-3.05)	*<0.001*	2.23 (1.48-3.34)	*<0.001*	2.06 (1.16-3.67)	*0.014*
>50	8.85 (6.50-12.06)	*<0.001*	7.00 (4.79-10.21)	*<0.001*	11.06 (6.51-18.79)	*<0.001*
**Gender**						
Male	Ref	*1.0*	Ref	*1.0*	Ref	*1.0*
Female	0.91 (0.83-1.00)	*0.042*	0.96 (0.85-1.08)	*0.490*	0.85 (0.74-0.98)	*0.026*
**Marital status**						
Married	Ref	*1.0*	Ref	*1.0*	Ref	*1.0*
Unmarried	1.04 (0.94-1.14)	*0.448*	1.09 (0.96-1.23)	*0.186*	0.97 (0.84-1.12)	*0.688*
Unknown	0.94 (0.77-1.13)	*0.499*	0.77 (0.59-1.01)	*0.061*	1.16 (0.89-1.50)	*0.273*
**Race**						
White	Ref	*1.0*	Ref	*1.0*	Ref	*1.0*
Black	1.06 (0.91-1.22)	*0.463*	1.33 (1.12-1.59)	*0.001*	0.72 (0.56-0.92)	*0.009*
Others	0.91 (0.76-1.08)	*0.268*	1.04 (0.84-1.29)	*0.726*	0.76 (0.57-0.99)	*0.045*
Unknown	0.25 (0.12-0.54)	*<0.001*	0.27 (0.10-0.73)	*0.010*	0.25 (0.08-0.78)	*0.017*
**Insurance**						
Insured	Ref	*1.0*	Ref	*1.0*	Ref	*1.0*
Uninsured	1.09 (0.85-1.40)	*0.492*	1.45 (1.09-1.93)	*0.010*	0.64 (0.40-1.02)	*0.058*
Unknown	1.67 (1.39-2.01)	*<0.001*	1.90 (1.51-2.38)	*<0.001*	1.29 (0.96-1.73)	*0.093*
**Primary Site**						
Bone	Ref	*1.0*	Ref	*1.0*	Ref	*1.0*
Soft tissue	1.42 (1.25-1.60)	*<0.001*	1.50 (1.28-1.77)	*<0.001*	1.28 (1.07-1.53)	*0.008*
**Location**						
Extremities	Ref	*1.0*	Ref	*1.0*	Ref	*1.0*
Head and neck	2.13 (1.86-2.44)	*<0.001*	2.13 (1.77-2.56)	*<0.001*	2.02 (1.65-2.46)	*<0.001*
Trunk	2.90 (2.59-3.26)	*<0.001*	3.03 (2.60-3.53)	*<0.001*	2.51 (2.12-2.98)	*<0.001*
Unknown	9.71 (8.26-11.42)	*<0.001*	10.38 (8.52-12.65)	*<0.001*	5.85 (4.61-7.42)	*<0.001*
**Grade**						
Grade I	Ref	*1.0*	Ref	*1.0*	Ref	*1.0*
Grade II	1.51 (1.08-2.09)	*0.015*	1.63 (0.99-2.66)	*0.054*	1.40 (0.91-2.16)	*0.126*
Grade III	4.78 (3.63-6.29)	*<0.001*	6.70 (4.45-10.10)	*<0.001*	3.09 (2.135-4.48)	*<0.001*
Grade IV	5.31 (4.07-6.93)	*<0.001*	6.47 (4.33-9.69)	*<0.001*	4.08 (2.87-5.79)	*<0.001*
Unknown	5.83 (4.49-7.56)	*<0.001*	8.13 (5.47-12.07)	*<0.001*	3.71 (2.63-5.23)	*<0.001*
**Laterality**						
Left-sided	Ref	*1.0*	Ref	*1.0*	Ref	*1.0*
Right-sided	0.84 (0.73-0.96)	*0.013*	0.77 (0.63-0.93)	*0.006*	0.94 (0.76-1.15)	*0.515*
One side, NOS	2.94 (2.63-3.30)	*<0.001*	3.04 (2.62-3.52)	*<0.001*	2.49 (2.10-2.95)	*<0.001*
Paired sides	4.11 (2.92-5.77)	*<0.001*	3.79 (2.45-5.84)	*<0.001*	3.75 (2.32-6.05)	*<0.001*
**Stage**						
Localized	Ref	*1.0*	Ref	*1.0*	Ref	*1.0*
Regional	2.39 (2.06-2.76)	*<0.001*	3.08 (2.49-3.80)	*<0.001*	1.84 (1.51-2.24)	*<0.001*
Distant	10.76 (9.51-12.17)	*<0.001*	16.39 (13.74-19.54)	*<0.001*	4.90 (4.13-5.81)	*<0.001*
Unknown	7.14 (6.07-8.41)	*<0.001*	8.85 (7.05-11.11)	*<0.001*	4.95 (3.97-6.18)	*<0.001*
**T stage**						
T1	Ref	*1.0*	Ref	*1.0*	Ref	*1.0*
T2	2.52 (2.16-2.95)	*<0.001*	3.61 (2.86-4.56)	*<0.001*	1.70 (1.38-2.09)	*<0.001*
T3	2.54 (1.27-5.08)	*0.008*	4.17 (1.78-9.73)	*0.001*	1.37 (0.43-4.36)	*0.596*
Unknown	7.81 (6.69-9.12)	*<0.001*	10.72 (8.50-13.51)	*<0.001*	4.69 (3.82-5.76)	*<0.001*
**N stage**						
N0	Ref	*1.0*	Ref	*1.0*	Ref	*1.0*
N1	3.21 (2.73-3.77)	*<0.001*	3.85 (3.18-4.68)	*<0.001*	2.06 (1.59-2.68)	*<0.001*
Unknown	4.01 (3.60-4.47)	*<0.001*	4.20 (3.66-4.82)	*<0.001*	3.07 (2.61-3.60)	*<0.001*
**Bone Met**						
No	Ref	*1.0*	Ref	*1.0*	Ref	*1.0*
Yes	5.33 (4.61-6.15)	*<0.001*	6.36 (5.38-7.52)	*<0.001*	2.85 (2.26-3.59)	*<0.001*
Unknown	3.62 (3.12-4.20)	*<0.001*	3.62 (3.00-4.36)	*<0.001*	2.98 (2.40-3.69)	*<0.001*
**Brain Met**						
No	Ref	*1.0*	Ref	*1.0*	Ref	*1.0*
Yes	9.71 (6.85-13.75)	*<0.001*	10.98 (7.58-15.91)	*<0.001*	3.78 (2.20-6.51)	*<0.001*
Unknown	3.33 (2.88-3.85)	*<0.001*	3.27 (2.73-3.92)	*<0.001*	2.79 (2.26-3.45)	*<0.001*
**Liver Met**						
No	Ref	*1.0*	Ref	*1.0*	Ref	*1.0*
Yes	7.65 (6.45-9.08)	*<0.001*	8.58 (7.07-10.40)	*<0.001*	3.81 (2.94-4.95)	*<0.001*
Unknown	3.64 (3.14-4.21)	*<0.001*	3.73 (3.11-4.47)	*<0.001*	2.87 (2.32-3.56)	*<0.001*
**Lung Met**						
No	Ref	*1.0*	Ref	*1.0*	Ref	*1.0*
Yes	6.12 (5.46-6.85)	*<0.001*	8.18 (7.14-9.38)	*<0.001*	2.81 (2.34-3.37)	*<0.001*
Unknown	4.43 (3.82-5.14)	*<0.001*	4.87 (4.04-5.87)	*<0.001*	3.21 (2.59-3.97)	*<0.001*
**Surgery**						
No Surgery	Ref	*1.0*	Ref	*1.0*	Ref	*1.0*
Local Surgery	0.09 (0.08-0.10)	*<0.001*	0.08 (0.07-0.10)	*<0.001*	0.14 (0.11-0.17)	*<0.001*
Partial Surgery	0.12 (0.09-0.15)	*<0.001*	0.10 (0.07-0.15)	*<0.001*	0.19 (0.13-0.26)	*<0.001*
Radical Surgery^*^	0.05 (0.05-0.06)	*<0.001*	0.05 (0.04-0.06)	*<0.001*	0.08 (0.06-0.10)	*<0.001*
Amputation	0.09 (0.07-0.13)	*<0.001*	0.10 (0.07-0.16)	*<0.001*	0.12 (0.07-0.19)	*<0.001*
Unknown	0.30 (0.23-0.40)	*<0.001*	0.39 (0.28-0.55)	*<0.001*	0.25 (0.15-0.41)	*<0.001*

*Radical excision or resection of lesion with limb salvage; Abbreviations: OR = Odds ratio; CI = Confidence interval; Ref = Reference.

**Table 3:** Multivariable logistic regression for analyzing the risk factors for early death in patients with bone and soft tissue tumor

Subject characteristics	Total early death		Cancer specific early death		Non-Cancer specific early death	
	OR (95% CI)	*P value*	OR (95% CI)	*P value*	OR (95% CI)	*P value*
**Age**						
≤18	Ref	*1.0*	Ref	*1.0*	Ref	*1.0*
19-50	3.17 (2.24-4.49)	*<0.001*	3.20 (2.10-4.87)	*<0.001*	2.61 (1.46-4.66)	*0.001*
>50	12.17 (8.80-16.84)	*<0.001*	9.00 (6.07-13.34)	*<0.001*	11.04 (6.44-18.90)	*<0.001*
**Gender**						
Male	Ref	*1.0*	-	*-*	Ref	*1.0*
Female	0.89 (0.80-0.99)	*0.033*	-	*-*	0.86 (0.74-1.00)	*0.044*
**Race**						
White	Ref	*1.0*	Ref	*1.0*	Ref	*1.0*
Black	1.08 (0.91-1.28)	*0.369*	1.36 (1.11-1.65)	*0.003*	0.75 (0.58-0.97)	*0.030*
Others	0.92 (0.75-1.13)	*0.425*	1.05 (0.82-1.34)	*0.707*	0.78 (0.59-1.05)	*0.097*
Unknown	0.29 (0.13-0.65)	*0.003*	0.31 (0.11-0.87)	*0.026*	0.30 (0.10-0.97)	*0.043*
**Insurance**						
Insured	Ref	*1.0*	Ref	*1.0*	-	*-*
Uninsured	1.05 (0.79-1.41)	*0.738*	1.32 (0.96-1.84)	*0.092*	-	*-*
Unknown	1.01 (0.80-1.28)	*0.953*	1.30 (0.98-1.73)	*0.065*	-	*-*
**Primary Site**						
Bone	Ref	*1.0*	Ref	*1.0*	Ref	*1.0*
Soft tissue	1.16 (1.00-1.35)	*0.053*	1.20 (0.99-1.45)	*0.064*	1.04 (0.85-1.27)	*0.705*
**Location**						
Extremities	Ref	*1.0*	Ref	*1.0*	Ref	*1.0*
Head and neck	1.08 (0.90-1.30)	*0.388*	1.09 (0.86-1.38)	*0.473*	1.10 (0.86-1.41)	*0.433*
Trunk	1.24 (1.04-1.47)	*0.015*	1.20 (0.96-1.50)	*0.107*	1.23 (0.98-1.55)	*0.078*
Unknown	1.39 (1.09-1.77)	*0.007*	1.30 (0.97-1.73)	*0.080*	1.24 (0.90-1.72)	*0.190*
**Grade**						
Grade I	Ref	*1.0*	Ref	*1.0*	Ref	*1.0*
Grade II	1.29 (0.91-1.82)	*0.157*	1.31 (0.79-2.19)	*0.298*	1.29 (0.83-2.01)	*0.256*
Grade III	3.29 (2.45-4.43)	*<0.001*	3.80 (2.47-5.84)	*<0.001*	2.35 (1.60-3.44)	*<0.001*
Grade IV	3.26 (2.45-4.34)	*<0.001*	3.22 (2.11-4.90)	*<0.001*	2.81 (1.95-4.04)	*<0.001*
Unknown	2.07 (1.56-2.74)	*<0.001*	2.49 (1.64-3.76)	*<0.001*	1.66 (1.15-2.38)	*0.006*
**Laterality**						
Left-sided	Ref	*1.0*	Ref	*1.0*	Ref	*1.0*
Right-sided	0.76 (0.65-0.89)	*0.001*	0.71 (0.58-0.87)	*0.001*	0.88 (0.72-1.09)	*0.251*
One side, NOS	1.37 (1.16-1.62)	*<0.001*	1.37 (1.11-1.70)	*0.003*	1.21 (0.96-1.51)	*0.101*
Paired sides	1.35 (0.90-2.04)	*0.152*	1.16 (0.70-1.91)	*0.563*	1.40 (0.83-2.36)	*0.212*
**Stage**						
Localized	Ref	*1.0*	Ref	*1.0*	Ref	*1.0*
Regional	1.71 (1.45-2.01)	*<0.001*	2.09 (1.66-2.62)	*<0.001*	1.37 (1.10-1.71)	*0.004*
Distant	2.78 (2.28-3.41)	*<0.001*	3.25 (2.51-4.20)	*<0.001*	2.02 (1.54-2.65)	*<0.001*
Unknown	1.40 (1.10-1.77)	*0.006*	1.57 (1.15-2.14)	*0.004*	1.35 (0.99-1.84)	*0.056*
**T stage**						
T1	Ref	*1.0*	Ref	*1.0*	Ref	*1.0*
T2	1.41 (1.18-1.68)	*<0.001*	1.88 (1.45-2.42)	*<0.001*	1.08 (0.86-1.36)	*0.491*
T3	0.67 (0.31-1.45)	*0.309*	1.04 (0.42-2.59)	*0.927*	0.54 (0.16-1.80)	*0.312*
Unknown	1.87 (1.54-2.28)	*<0.001*	2.21 (1.680-2.90)	*<0.001*	1.43 (1.11-1.84)	*0.006*
**N stage**						
N0	Ref	*1.0*	Ref	*1.0*	Ref	*1.0*
N1	1.03 (0.86-1.26)	*0.738*	1.12 (0.90-1.40)	*0.323*	0.91 (0.68-1.21)	*0.495*
Unknown	1.10 (0.93-1.30)	*0.273*	1.12 (0.92-1.37)	*0.262*	1.01 (0.80-1.26)	*0.962*
**Bone Met**						
No	Ref	*1.0*	Ref	*1.0*	Ref	*1.0*
Yes	1.09 (0.90-1.33)	*0.379*	1.22 (0.99-1.51)	*0.066*	0.85 (0.65-1.13)	*0.260*
Unknown	0.90 (0.50-1.62)	*0.714*	0.82 (0.43-1.57)	*0.549*	1.21 (0.56-2.58)	*0.631*
**Brain Met**						
No	Ref	*1.0*	Ref	*1.0*	Ref	*1.0*
Yes	2.10 (1.38-3.20)	*0.001*	2.03 (1.33-3.11)	*0.001*	1.21 (0.67-2.16)	*0.530*
Unknown	0.75 (0.39-1.44)	*0.384*	0.69 (0.34-1.40)	*0.308*	1.01 (0.43-2.37)	*0.985*
**Liver Met**						
No	Ref	*1.0*	Ref	*1.0*	Ref	*1.0*
Yes	0.94 (0.76-1.16)	*0.547*	1.00 (0.80-1.27)	*0.976*	0.80 (0.59-1.09)	*0.155*
Unknown	1.25 (0.69-2.26)	*0.457*	1.45 (0.78-2.71)	*0.243*	0.78 (0.35-1.70)	*0.525*
**Lung Met**						
No	Ref	*1.0*	Ref	*1.0*	Ref	*1.0*
Yes	1.44 (1.20-1.73)	*<0.001*	1.82 (1.49-2.23)	*<0.001*	0.78 (0.61-1.01)	*0.059*
Unknown	1.53 (0.98-2.41)	*0.064*	1.65 (1.01-2.70)	*0.044*	1.08 (0.59-1.96)	*0.805*
**Surgery**						
No Surgery	Ref	*1.0*	Ref	*1.0*	Ref	*1.0*
Local Surgery	0.19 (0.16-0.23)	*<0.001*	0.24 (0.19-0.30)	*<0.001*	0.22 (0.17-0.27)	*<0.001*
Partial Surgery	0.24 (0.18-0.31)	*<0.001*	0.26 (0.18-0.39)	*<0.001*	0.28 (0.20-0.40)	*<0.001*
Radical Surgery^*^	0.11 (0.09-0.13)	*<0.001*	0.14 (0.11-0.18)	*<0.001*	0.12 (0.09-0.16)	*<0.001*
Amputation	0.19 (0.14-0.27)	*<0.001*	0.25 (0.16-0.39)	*<0.001*	0.19 (0.12-0.31)	*<0.001*
Unknown	0.36 (0.27-0.49)	*<0.001*	0.54 (0.38-0.77)	*0.001*	0.27 (0.17-0.45)	*<0.001*

*Radical excision or resection of lesion with limb salvage; Abbreviations: OR = Odds ratio; CI = Confidence interval; Ref = Reference.

## Abbreviations

SEER: Surveillance, Epidemiology, and End Results
ROC: receiver operating characteristics
AUC: area under the curve
OR: Odds ratio
CI: Confidence interval
Ref: Reference
NA: Not applicable

## References

[1] Siegel RL, Miller KD, Jemal A (2019). Cancer statistics, 2019. CA Cancer J Clin.

[2] Trautmann F, Schuler M, Schmitt J (2015). Burden of soft-tissue and bone sarcoma in routine care: Estimation of incidence, prevalence and survival for health services research. Cancer Epidemiol.

[3] Kollar A, Rothermundt C, Klenke F (2019). Incidence, mortality, and survival trends of soft tissue and bone sarcoma in Switzerland between 1996 and 2015. Cancer Epidemiol.

[4] Pingping B, Yuhong Z, Weiqi L (2019). Incidence and Mortality of Sarcomas in Shanghai, China, During 2002-2014. Front Oncol.

[5] Davenport JR, Vo KT, Goldsby R (2016). Conditional Survival and Predictors of Late Death in Patients With Ewing Sarcoma. Pediatr Blood Cancer.

[6] Wang B, Tu J, Yin J (2015). Development and validation of a pretreatment prognostic index to predict death and lung metastases in extremity osteosarcoma. Oncotarget.

[7] Nandra R, Hwang N, Matharu GS (2015). One-year mortality in patients with bone and soft tissue sarcomas as an indicator of delay in presentation. Ann R Coll Surg Engl.

[8] Penel N, Glabbeke MV, Mathoulin-Pelissier S (2011). Performance status is the most powerful risk factor for early death among patients with advanced soft tissue sarcoma: the European Organisation for Research and Treatment of Cancer-Soft Tissue and Bone Sarcoma Group (STBSG) and French Sarcoma Group (FSG) study. Br J Cancer.

[9] Blakey K, Feltbower RG, James PW (2018). Socio-economic patterning in early mortality of patients aged 0-49 years diagnosed with primary bone cancer in Great Britain, 1985-2008. Cancer Epidemiol.

[10] Sun YP, Wang X, Gao YS (2017). Primary cardiac sarcoma complicated with cerebral infarction and brain metastasis: A case report and literature review. Cancer Biomark.

[11] Khan H, Chaubey S, Edlin J (2014). Primary cardiac synovial sarcoma. A rare tumor with poor prognosis. Asian Cardiovasc Thorac Ann.

[12] Tsuchie H, Emori M, Nagasawa H (2019). Prognosis of Primary Osteosarcoma in Elderly Patients: A Comparison between Young and Elderly Patients. Med Princ Pract.

[13] Kumar R, Kumar M, Malhotra K (2018). Primary Osteosarcoma in the Elderly Revisited: Current Concepts in Diagnosis and Treatment. Curr Oncol Rep.

[14] Karski EE, Matthay KK, Neuhaus JM (2013). Characteristics and outcomes of patients with Ewing sarcoma over 40 years of age at diagnosis. Cancer Epidemiol.

[15] Callegaro D, Miceli R, Bonvalot S (2019). Development and external validation of a dynamic prognostic nomogram for primary extremity soft tissue sarcoma survivors. EClinicalMedicine.

[16] Aizer AA, Chen MH, McCarthy EP (2013). Marital status and survival in patients with cancer. J Clin Oncol.

[17] Penumarthy NL, Goldsby RE, Shiboski SC (2020). Insurance impacts survival for children, adolescents, and young adults with bone and soft tissue sarcomas. Cancer Med.

[18] Smeland S, Bielack SS, Whelan J (2019). Survival and prognosis with osteosarcoma: outcomes in more than 2000 patients in the EURAMOS-1 (European and American Osteosarcoma Study) cohort. Eur J Cancer.

[19] Gotzl R, Sterzinger S, Semrau S (2019). Patient's quality of life after surgery and radiotherapy for extremity soft tissue sarcoma - a retrospective single-center study over ten years. Health Qual Life Outcomes.

[20] Oh E, Seo SW, Han KJ (2018). A Longitudinal Study of Functional Outcomes in Patients with Limb Salvage Surgery for Soft Tissue Sarcoma. Sarcoma.

[21] Wang X, Mao M, Xu G (2019). The incidence, associated factors, and predictive nomogram for early death in stage IV colorectal cancer. Int J Colorectal Dis.

